# Successful treatment for distal-arch aortic aneurysm in a cold agglutinin-positive patient via physician-modified thoracic endovascular aortic repair: a case report

**DOI:** 10.1186/s44215-025-00195-5

**Published:** 2025-07-28

**Authors:** Rika Oshima, Tetsuya Sato, Ryotaro Yamada, Takuya Kawahara, Riki Sumiyoshi, Kosuke Miyoshi, Kazunori Hashimoto, Kenichi Hashizume, Satoshi Itoh

**Affiliations:** 1 Center for Medical Education and Training, Yokohama City Minato Red Cross Hospital, Yokohama, Japan; 2 Department of Cardiovascular Surgery, Yokohama City Minato Red Cross Hospital, 3-12-1 Shinyamashita, Naka-Ku, Yokohama, Kanagawa Japan; 3 Department of Cardiovascular Surgery, Saiseikai Utsunomiya Hospital, Tochigi, Japan

**Keywords:** Aneurysm, Physician-modified endograft, Cold agglutinin

## Abstract

**Background:**

Cold agglutinin disease (CAD) is sometimes incidentally detected before cardiovascular surgery. Several methods to prevent complications associated with CAD after cardiac surgery have been reported, but there are no reports of the use of physician-modified TEVAR to date.

**Case presentation:**

A 76-year-old man with an arch aortic saccular aneurysm was scheduled to undergo arch aortic replacement. However, cold agglutinin syndrome was incidentally detected before open heart surgery. The safety of cardiopulmonary surgery under hypothermia for patients with cold agglutinin disease is unknown, as intravascular hemolysis is a source of concern for patients sensitive to cold stimulation. Instead, we performed physician-modified thoracic endovascular aortic repair (3 fenestrations and 1 branch), as the aneurysm in this case was suitable for thoracic endovascular aortic repair (TEVAR). As a result, the patient recovered well without any complication.

**Conclusions:**

The long-term prognosis of physician-modified thoracic endovascular aortic repair remains unclear, and its use is limited to high-risk patients who require open chest surgery. Also, the impact of cold agglutination on stent grafts in CAD patients has not been reported. Despite that situation, this case illustrated that physician-modified TEVAR can be safely performed without significant postoperative complications, such as coagulation-fibrinolytic abnormalities or embolic events. Further studies are needed to establish the indications for this procedure in CAD patients.

## Background

There are no reports on the safety of open thoracotomy under moderate hypothermic circulatory arrest in patients with cold agglutinin syndrome. It is not known how hypothermic circulatory arrest affects agglutinin; thus, open thoracotomy should be avoided whenever possible. Here, we report the efficacy of physician-modified TEVAR for aortic arch aneurysms in a patient with cold agglutinin syndrome to avoid hypothermic circulatory arrest. The local medical technical ethics committee approved the study. Written informed consent was obtained from the patient and his family.

## Case presentation

**Fig. 1:**
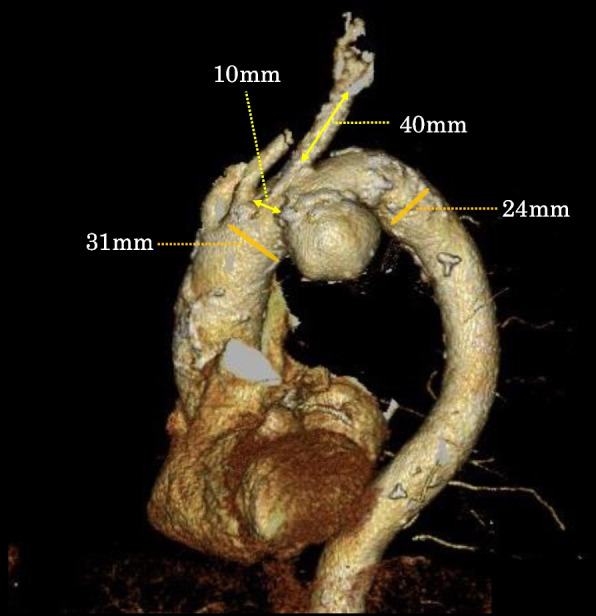
Measurements for physician-modified TEVAR.There was almost no distance from the origin of the left subclavian artery to the aneurysm, and the 3-dimensional distance from the origin of the left common carotid artery to the aneurysm was 10 mm. The diameter of the left subclavian artery was 10 mm, the distance from the origin of the left subclavian artery to the bifurcation of the vertebral artery was 40 mm, the diameter of the aortic neck was 31 mm, and the diameter of the descending aorta was 24 mm

The patient was a 76-year-old man with sigmoid colon cancer and a distal arch aortic aneurysm. The saccular aneurysm protruded into the lesser curvature of Zones 2–3 of the aortic arch and had a maximum short diameter of 54 mm. Since TEVAR is unsuitable owing to the shape and location of the aneurysm, aortic arch replacement was planned. A blood transfusion study was performed preoperatively and revealed an abnormality in the blood group system. A blood test revealed that the patient’s blood was positive for type A antigens, which has not only B-type antibodies but also A-type antibodies. Therefore, the results of the test were inconclusive. When the blood was preheated to 37℃, the cold agglutinin was removed, erythrocyte agglutination disappeared, and anti-A antibodies undetected (Table 1). The titer of cold agglutinins was 16,384 times the normal value, thus the patient was diagnosed with CAD (Table 2). Therefore, we decided not to perform the aortic arch replacement because the replacement procedure needs hypothermic circulatory arrest and therefore potentially unsafe for patients with cold agglutination. We remeasured the aortic arch aneurysm for TEVAR and noted that there was practically no distance between the origin of the left subclavian artery and the aneurysm, the 3-dimensional distance between the origin of the left common carotid artery and the aneurysm was 10 mm, the diameter of the left subclavian artery was 10 mm, the distance from the origin of the left subclavian artery to the bifurcation of the vertebral artery was 40 mm, the diameter of the aortic neck was 31 mm, and the diameter of the descending aorta was 24 mm (Fig. 1). On the basis of these measurements, we considered TEVAR feasible and scheduled physician-modified TEVAR.

**Table 1:** Transfusion tests

	Non-preheated	Preheated	After removing Cold Agglutinin
**ABO Blood Group System**			
** Cell**			
Anti-A Antibody	4 +	4 +	4 +
Anti-B Antibody	No Agglutination	No Agglutination	No Agglutination
** Serum**			
Type A Antigen	3 +	No Agglutination	No Agglutination
Type B Antigen	3 +	1 +	2 +
** Coombs Test**			
Direct Coombs Test	Negative	-	-
Indirect Coombs Test	Negative	-	-

A blood test revealed that the patient's blood was positive for type A antigens, anti-A and anti-B antibodies. As this result was inconclusive, the blood was preheated to 37℃ or the cold agglutinin was removed to determine the true type of blood. After preheating and removing cold agglutinin, anti-A antibodies were undetected, which means the patient's blood is type A

**Table 2:** Pre- and postoperative laboratory data

		Preoperative	Postoperative				
			Day2	Day9	Day76	Unit	Reference Value
**Complete Blood Count**							
WBC		4000	8400^a^	6400	3500	/μL	3900–9800
RBC		354	347^a^	308	428	10^4^/μL	427–570
Hb		12	11.6^a^	9.9	13.1	g/dL	13.5–17.6
Ht		34.3	33.2^a^	29.5	39.5	%	39.8–51.8
Ret		24	-	-	-		5–20
PLT		21.1	16.7^a^	38	19.8^a^	10^4^/μL	13.1–36.2
**Serum Chemistries**							
CRP		0.1	2.8	4.3	0	mg/dL	0–0.3
T-Bil		0.5	1.5	0.7	0.5	mg/dL	0.2–1.0
Na		142	139	138	139	mEq/L	136–147
K		3.9	3.7	3.6	4.3	mEq/L	3.6–5.0
Cl		107	105	104	104	mEq/L	99–109
AST		26	33	19	30	U/L	10–40
ALT		21	20	13	18	U/L	5–40
LDH		173	203	203	220	U/L	124–222
**Coagulation Studies**							
PT		11.3	11.8	12.3	-	Sec	10.5–13.5
PT-INR		0.98	1.02	1.07	-	%	-
APTT		27.7	29.3	-	-	Sec	25–35
D-dimer		0.8	-	-	-	μg/mL	0–1.0
**Cold Agglutination Titer**		16384	-	-	8192	times	< 64

Preoperatively, the patient was mildly anemic, with high reticulocyte counts and cold agglutination titers. Anemia progressed until postoperative day 9, but recovered by day 76. Cold agglutinin titer decreased but remained high. During the course, the coagulation-fibrinolytic system data did not change significantly

^a^Results of re-inspection after heating

**Fig. 2:**
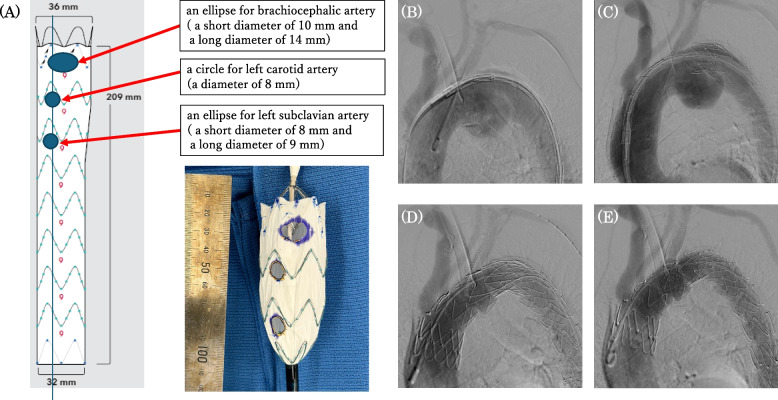
Intraoperative images. **A** The blueprint and actual fenestrated stent graft created on the basis of preoperative measurements. **B** The initial contrast image before stent placement. **C** The first stent graft (Zenith Alpha® Thoracic Endovascular Graft: ZTA-P-28–155) was implanted from Zone 3. **D** The second fenestrated stent graft (Zenith Alpha® Thoracic Endovascular Graft: ZTA- PT-36–32–209) was implanted from Zone 0 to the descending aorta. **E** Viabahn® (diameter,13 mm; length,50mm) was inserted into the left subclavian artery using the pull-through technique and deployed so that it would not interfere with the vertebral artery. The final contrast image showed no leakage

**Fig. 3:**
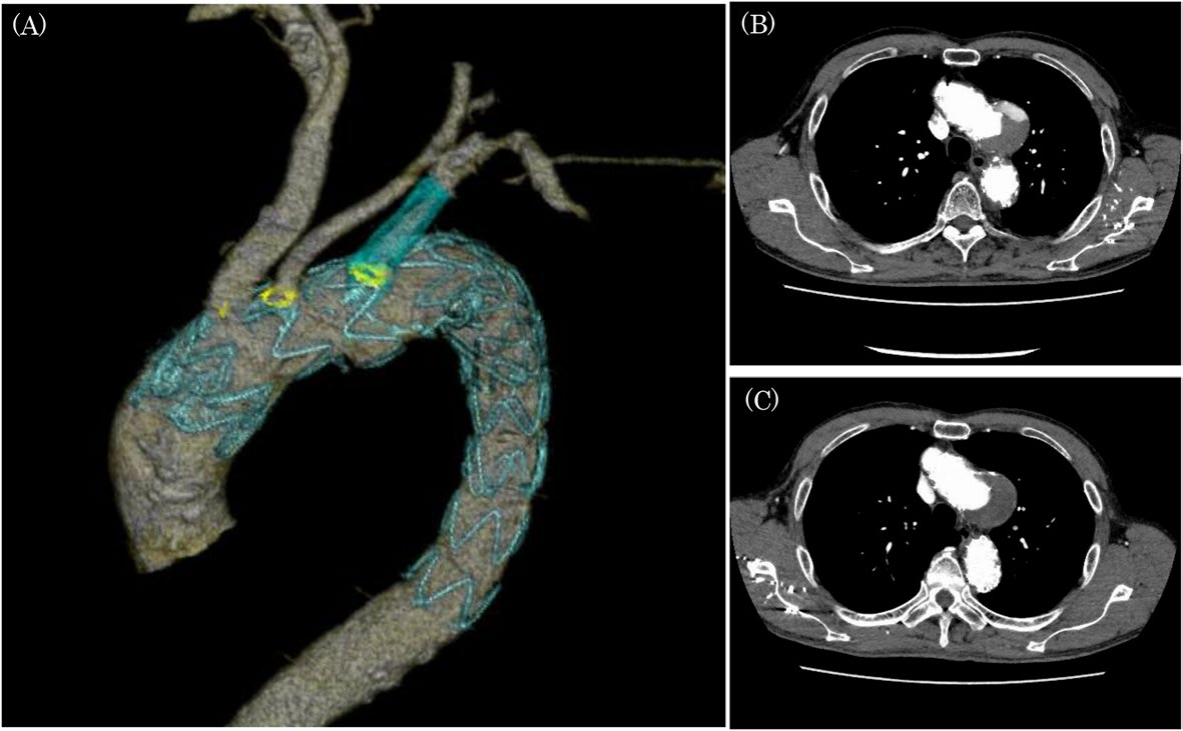
Postoperative computed tomography images. **A** 3D CT image on the 4th postoperative day. The position of the fenestration completely matched the cervical branch (yellow), and the vertebral artery was preserved. **B** Contrast CT on postoperative day 4: a type 3a endoleak was suspected at the Viabahn® implantation site. **C** Contrast CT at 3 months after surgery: the endoleak had disappeared after continuous treatment with 1500 mg of tranexamic acid per day

The Zenith Alpha® Thoracic Endovascular Graft system(William Cook Europe ApS, Denmark) was used for physician-modified TEVAR (Fig. 2). A segment of the aortic stent graft was unsheathed, and the barb at the first stent was cut with nippers. Fenestrations were made at the CT-measured locations of the three arterial origins from the aortic arch and were created with an Accu-Temp® cautery. The edges of the fenestrations were marked with gold-plated tungsten loops of Amplatz Goose Neck® Snares. The stent graft was resheathed by bending with 10 mm silk tape. The sizes of the fenestrations were an ellipse with a short diameter of 10 mm and a long diameter of 14 mm for the brachiocephalic artery, an 8 mm diameter circle for the left common carotid artery, and an ellipse with a short diameter of 8 mm and a long diameter of 9 mm for the left subclavian artery. A guide wire was passed through the right common femoral artery to the ascending aorta. A Zenith Alpha® Thoracic Endovascular Graft (ZTA-P-28–155) was deployed at the descending aorta. A physician-modified Zenith Alpha® Thoracic Endovascular Graft (ZTA-PT-36–32–209) was subsequently deployed from Zone 0 with controlled pacing at 110 bpm. Oversize ratios of landings were 16.1% at the proximal and 33.3% at the distal. Next, the guide wire was passed through the left branchial artery to the right femoral artery via endograft fenestration for the left subclavian artery. A Viabahn® stent (diameter, 13 mm; length, 50mm, W. L. Gore, Newark, DE, USA) was deployed into the left subclavian artery over the pull-through wire, which preserve the vertebral arterial flow. The physician-modified TEVAR was completed without contrast agent leakage. On postoperative contrast CT, a Type 3a endoleak from the site of Viabahn® stent implantation was suspected, and the patient was started on 1500 mg of tranexamic acid per day. Three months later, contrast-enhanced CT revealed that the endoleak in the aneurysm had disappeared, and the aneurysm diameter was 54 mm and unchanged (Fig. 3).

## Discussion and conclusion

Cold agglutinin disease (CAD) is an autoimmune hemolytic anemia, in which erythrocyte aggregation and extravascular hemolysis are caused by antibodies called cold agglutinins, which are among the cold-type antibodies that recognize antigens on red blood cells at temperatures below normal body temperature. A retrospective review of cases in Nordic countries revealed that the estimated incidence of CAD was approximately 1–180 per million and that the prevalence of CAD was approximately 13–16 per million, with a male–female ratio of 0.55, a mean age at onset in the mid-to-late 60s, and a predominance in individuals between 30 and 90 years old [1, 2]. As cardiovascular surgery is commonly performed under hypothermic circulatory arrest, preoperative cold coagulant screening is performed in some centers. A report on the usefulness of cold coagulant screening before cardiopulmonary bypass surgery revealed that only 0.3% of patients were cold agglutinin positive [3]. Although rare, CAD can cause serious complications such as stroke during cardiovascular surgery and should be identified before surgery. The following three criteria are generally used to diagnose CAD: the presence of hemolytic anemia (a median Hb level of 9.5 g/dL), a high reticulocyte count, high LDH level, high indirect bilirubin level, low haptoglobin level, positive direct Coombs test for C3d only or C3d and weak IgG in some cases, and a cold agglutination titer greater than 64 at 4℃ [4, 5]. The causes of secondary CAD include EBV infection and mycoplasma infection, autoimmune diseases such as SLE, lymphoid malignancies such as non-Hodgkin's lymphoma, Waldenström’s macroglobulinemia, and lymphoproliferative diseases. Therefore, when CAD is suspected before surgery, it is important to consider these diseases [6, 7]. It has also been reported that solid tumors are significantly more common in patients with CAD than in patients with warm AIHA, thus searching for malignancy at the time of diagnosis of AIHA, including CAD, is needed [8]. In this case, the patient did not have anemia or elevated hemolytic markers as described above (Table 2), and he was unaware of any cold-induced symptoms. Therefore, the patient was not known to have CAD until the preoperative transfusion test was conducted. After preheating to 37℃ or removing cold agglutinin, erythrocyte agglutination disappeared. CAD was subsequently diagnosed on the basis of a titer of cold agglutinin 16,384 times the nomal value. Since additional measurements of soluble IL-2 receptor and IgM were negative for clonally proliferating hematologic disease, the patient was diagnosed with primary CAD. Treatment options for open-heart surgery for primary CAD includes preoperative plasma exchange, anti-complement monoclonal antibody preparations, and cardiopulmonary bypass without hypothermia [9, 10]. However, there have been no reports on the management of CAD during aortic surgery under hypothermic circulatory arrest. Cold agglutinins are often high-molecular-weight IgM and can be effectively removed via plasma exchange. Because cardiopulmonary procedures are generally performed under hypothermia to protect the brain, preoperative plasma exchange may be performed to reduce the number of cold agglutinins in circulation. This approach is expected to prevent intraoperative hemolysis and cold-induced erythrocyte aggregation, but its effectiveness has never been verified. Although plasma exchange can eliminate IgM, it cannot decrease IgM production itself and should be considered a temporary measure. In addition, only case reports have revealed that the administration of anti-complement monoclonal antibody preparations such as eculizumab or sutimlimab reduces intravascular hemolysis. Although there is a report of a case in which mild hypothermia at 34℃ was the target for cardiopulmonary surgery using room temperature cardioplegia solution [11], there is no follow-up report on the long-term prognosis or the incidence of cerebrovascular events, and its safety has not yet been established.

We chose other than the above methods instead of open-heart surgery using an artificial heart and lung under hypothermia. Currently, for patients for whom standard TEVAR is anatomically challenging, chimney stents, debranching TEVAR, and physician-modified (branched) TEVAR are considered. Debranching TEVAR is generally selected for patients at high risk for complications after open thoracotomy. Compared with open surgical aortic arch repair without the frozen elephant trunk procedure, debranching TEVAR is associated with shorter intensive care unit and hospital stays, a lower postoperative blood transfusion volume, and a lower reoperation rate due to hemorrhagic complications. Still, it is associated with higher incidences of spinal cord ischemia and stroke than surgical repair is [12]. In addition, comparisons of physician-modified TEVAR and branching TEVAR revealed that the operative duration of the former is shorter, the mortality rate is lower, and their rates of patency, perioperative complications, and endoleaks are comparable [13, 14].

We considered that physician-modified TEVAR was the most suitable treatment option for this case, and the risk of stroke in this case was not high since there was little calcification and arteriosclerosis in the landing zone. The anatomical indications for physician-modified TEVAR at our hospital are as follows: 1) good landing zone characteristics and a distance of at least 2 cm from the main branch in three dimensions, 2) a distance of at least 3 cm from the origin of the left subclavian to the vertebral artery, and 3) mild tortuosity of the arch to the descending aorta and easy predictability of the branch location. Recently, 3D printing technologies have made it possible to predict the exact location of the branch preoperatively, and anatomical indications are expected to be further expanded in the future [15, 16].

In this case, the Zenith Alpha® device was selected instead of NAJUTA® which is a specialized device approved in Japan for distal-arch aortic aneurysms. The semi-custom-made fenestrated stent graft NAJUTA® utilizes 3D vascular modeling, but its use can be limited by specific anatomical and procedural considerations. The NAJUTA® device is designed with a recommendation to maintain a 20 mm distance from the partial jugular branch. In this case, where this distance is 10 mm, the device was not a suitable choice. Additionally, NAJUTA® is not well-suited for additional interventions targeting endoleaks at fenestration sites, as the internal skeleton of the stent obstructs the placement of an additional stent.

In addition, there was type 3 endoleak in this case. Due to the limitations of the fitting of the polyester fabric of Zenith Alpha® and the Viabahn®, some leakage was expected. It has been reported that type 3 endoleak occurred in 9.5 % of cases and additional treatment was required in 7.1 % [17]. As in this case, the endoleak may disappear as the long-term result, and careful follow-up is necessary. If the endoleak persists, lining the Viabahn® stent with a bare metal stent may be effective.

Compared with that of other stent grafting procedures, the long-term prognosis of physician-modified TEVAR performed in this study is still unknown, and only patients considered at high risk for complications associated with open chest surgery are recommended for the procedure. Besides, the effect of cold agglutination on implanted stent grafts in CAD patients has not been reported. However, in this case, we demonstrated that physician-modified TEVAR can be performed safely without postoperative adverse events such as abnormalities of the coagulation-fibrinolytic system or embolism. Further case series are needed to determine the scope of indications for physician-modified TEVAR and the choice of procedure for CAD patients.

## Data Availability

Not applicable.

## Abbreviations

CAD: Cold agglutinin disease
TEVAR: Thoracic endovascular aortic repair
AIHA: Autoimmune hemolytic anemia
